# Community-level Factors and their Associations with Changing Opioid Overdose Fatality Rates in Kentucky, 2019–2021

**DOI:** 10.13023/jah.0601.07

**Published:** 2024-09-01

**Authors:** Shawn R. Nigam, Philip M. Westgate, Svetla Slavova, Rachel Vickers-Smith, Katherine L. Thompson

**Affiliations:** University of Kentucky; University of Kentucky; University of Kentucky; University of Kentucky; University of Kentucky

**Keywords:** Appalachia, generalized estimating equations, negative binomial regression, opioid use disorder, OUD, overdose

## Abstract

**Introduction:**

Kentucky has one of the highest opioid overdose fatality rates in the United States, which has increased significantly from 2019 to 2020. The COVID-19 pandemic has caused lasting effects on mental health and health care, which have been linked with increased opioid overdose. These effects are exacerbated in Appalachian regions, where there is a lack of sufficient access to community pharmacies and adequate health care.

**Purpose:**

In this study, we characterize changes in opioid overdose fatality rates in Kentucky from 2019 to 2021, with a specific focus on changes in Appalachian vs non-Appalachian counties. We aim to identify associations between community-level factors and opioid overdose fatality rates and how such associations may have changed from 2019 to 2021.

**Methods:**

County-level data were used to fit a marginal GEE-type negative binomial model to determine factors associated with opioid overdose fatality rates in 2019 (before the COVID-19 pandemic) and 2021 (during the COVID-19 pandemic).

**Results:**

Opioid overdose fatality rates increased from 2019 to 2021 (Rate Ratio: 1.82). This increase was much larger in adjacent-to-metropolitan (RR: 2.54) and Appalachian (RR: 2.38) counties. Age was associated with opioid overdose fatality rates in both 2019 and 2021, and the observed association for age was stronger in 2021. Appalachian status was associated with opioid overdose fatality rates in 2021. Metropolitan county status was associated with opioid overdose fatality rates in 2019, whereas adjacent-to-metropolitan county status was associated with opioid overdose fatality rates in 2021.

**Implications:**

Adjacent-to-metropolitan status’s association with overdose fatalities in 2021 may indicate a differential effect of COVID-19 on suburban communities. Future studies should investigate additional factors related to COVID-19 as well the lasting effects of the pandemic on the landscape of opioid overdose in Kentucky.

## INTRODUCTION

Drug overdose remains a leading cause of death in the U.S., with over 107,000 deaths in 2021.^1^ Of these deaths, almost three-quarters can be attributed to opioids.^2^ Opioid overdose deaths have increased dramatically, with nationwide rates doubling between 2010 and 2019.^3^ Initially, the majority of these deaths resulted from prescription opioid overdose. However, following a number of initiatives at state and national levels to reduce prescription opioid overprescribing, misuse and diversion (e.g., reformulation of OxyContin as an abuse-deterrent formulation, strengthening state prescription drug monitoring program laws, closure of “pill-mill” clinics, improved prescriber education, and mandated reduction in prescription opioid production by the Drug Enforcement Administration), prescription opioid overdose deaths decreased significantly in 2012–2013.^4^,^5^ Yet without adequate capacity for opioid use disorder (OUD) treatment, demand for opioids continued; alongside this shift, heroin became cheaper and more accessible, resulting in an increase in heroin-related overdose deaths post-2011. In 2014–2015, illicitly produced fentanyl and fentanyl analogs became more widely available, and have proven to be much deadlier than prescription opioids because of their potency.^6^ A study by Gladden et al., conducted in 27 states, demonstrated a 426% increase from 2013 to 2014 in drug products obtained by law enforcement that tested positive for fentanyl.^7^ Since 2016, fentanyl—either mixed with other opioids or marketed as “heroin”—has been the largest contributor to opioid-related deaths, causing almost 50% of them.^8^ Though there are people who willingly seek out fentanyl, many who overdose consume fentanyl unknowingly. Thus, both the potency and initial inconspicuousness of fentanyl have contributed to the increase in opioid overdose deaths in recent years.

Kentucky is especially affected by the opioid overdose epidemic, having the fifth-highest opioid overdose fatality rate among U.S. states in 2020.^9^ Drug overdose has long been an issue in Kentucky, as prescription opioids such as oxycodone have been prevalent in rural areas due to aggressive marketing and lack of access to alternative pain treatment.^10^ In addition, there is a relatively high prevalence of physical labor occupation in Appalachian Kentucky, which is frequently associated with acute and chronic pain.^10^ The overprescribing of opioid analgesics is a contributing factor towards the high rate of opioid use in Kentucky.^11^ Prior prescription opioid misuse is linked to the use of heroin and illicit fentanyl, and the decrease in the availability of diverted prescription opioids has caused much of the opioid misuse in Kentucky to transition towards these forms of opioids.^7^,^12^,^13^ Despite legislation in the mid-2010s aimed at increasing the availability of the opioid antagonist naloxone, as well as substance use disorder treatment services, fentanyl overdose death rates have increased significantly in recent years.^12^,^14^,^15^ Factors such as poverty, unemployment, and family stressors have been linked to opioid overdose in Kentucky communities, especially rural communities.^16^,^17^ Opioid misuse in remote rural communities poses additional risk, as offering help in these areas is much harder due to lack of cellular phone service and long distances from healthcare providers. These socioeconomic root factors make tackling the opioid overdose problem in Kentucky much more complex.

In recent years, the epidemic has intensified. Nationwide, synthetic opioid overdose deaths increased by 39% from the 12-month period ending in May 2020 to the 12-month period ending in May 2021.^1^ From 2019 to 2020, opioid overdose death rates among Kentucky residents increased from 23.10 to 35.74 per 100,000 residents, a 55% increase, with fentanyl overdose deaths increasing from 17.08 to 30.10 per 100,000 residents, a 76% increase.^15^ There was a large increase in opioid overdoses in Kentucky during the early stages of the COVID-19 pandemic; emergency medical service calls for opioid overdose in Kentucky rose by 17% from the 2-month period prior to the pandemic to the first two months within it (March and April 2020).^18^ However, it is still unclear which factors have been the most impactful in increasing opioid overdose fatalities after the onset of COVID-19.

There have been several societal changes that have occurred due to COVID-19 that have been identified as potential links to this increase.^19^ Of these changes, there are two main factors—mental health and unemployment—that the present study investigates. Stay-at-home orders at the beginning of the COVID-19 pandemic have been linked with increased psychological stress, which has been associated with economic instability and social isolation.^20^ Of all U.S. adults, 13.9% reported symptoms of psychological distress during the early stages of the COVID-19 pandemic (April 2020) compared to 3.9% in 2018.^21^ An increase in substance use during the COVID-19 pandemic may also be indicative of stressors causing individuals to turn to substances such as opioids for relief, thus increasing their risk of substance-use-related harm.^22^ In addition, access to mental health care was stymied during the pandemic, largely due to closures of clinician offices and concerns about in-person care.^20^ The lack of mental health care is uniquely concerning in the wake of COVID-19 due to the increased need for treatment in an environment with intensified psychological stressors.^23^

The pandemic also caused drastic changes in unemployment in the U.S., especially during its initial stages. The unemployment rate tripled from February to April 2020 and did not return to pre-COVID-19 levels until September 2021.^24^ Increases in unemployment rates have been found to be associated with increased levels of opioid overdose.^25^,^26^ More generally, unemployment has been found to be associated with adverse health behaviors, such as substance misuse.^27^ Unemployment introduces economic stressors that can limit access to health care as well as inhibit the ability to adhere to substance misuse treatment regimens.^28^,^29^ In short, the rapid and severe increase in unemployment during the COVID-19 pandemic has introduced economic stressors that may cause individuals to turn to opioid misuse, often resulting in harmful situations that increase likelihood of overdose deaths.

Several demographic factors have been found relevant to changes in opioid overdose mortality trends. Age, for example, is important to account for, as opioid overdose is generally more common in younger populations.^30^ Race is another factor associated with disparity in drug overdose deaths.^31^ Specifically, the average annual percentage change in opioid overdose fatalities between 2013 and 2020 was nearly twice as high among black Americans compared to white Americans (26.16 v. 13.19).^32^ In addition, there is evidence that black communities were uniquely affected by COVID-19 due to factors such as access to health care.^33^ Disparities in socioeconomic status and poverty within communities have been linked to increased risk of opioid overdose due to lack of access to health care as well as the prevalence of manual labor.^26^,^34^,^35^ Another factor related to both socioeconomic status and the COVID-19 pandemic is access to health insurance. During the initial stages of the pandemic (the three-month period in 2020 between April to July), the percentage of uninsured individuals increased by 1.4%.^36^ Access to health insurance is crucial for opioid use disorder treatment, as services, such as Medicaid, cover medication for opioid use disorder (MOUD).^37^

In addition to access to health insurance, availability of MOUD for opioid overdose is crucial. Both methadone and buprenorphine have been shown to be effective compared to other OUD treatment pathways.^38^ Methadone is an opioid agonist that must be administered at certified opioid treatment programs (OTPs).^39^ Because of geographic and policy limitations, access to methadone treatment from OTPs is often difficult, which was exacerbated during COVID-19.^40^,^41^ Access to methadone treatment is more difficult in rural areas, where distance to OTPs is much greater, on average.^42^ Buprenorphine is a partial opioid agonist that can be prescribed or administered by certain trained healthcare practitioners.^43^ Access to buprenorphine is disproportionately limited in rural areas, with many rural counties not having access to a provider.^44^ During the initial months of the COVID-19 pandemic, both overall buprenorphine prescriptions and Kentucky transmucosal (TM) buprenorphine reception rates decreased significantly.^45^,^46^ Recent initiatives, such as allowing prescription without an in-person physician meeting, have been introduced to counteract this disruption to buprenorphine access.^20^,^47^

Naloxone is a life-saving opioid antagonist that is available in community pharmacies. The U.S. Surgeon General called naloxone a key part of the public health response to the opioid epidemic.^48^ While there have been recent efforts to expand naloxone distribution in Kentucky through legislation and pharmacist training programs, barriers to naloxone access remain in rural communities due to scarcity of accessible community pharmacies as well as a lack of knowledge of the availability of naloxone.^49^,^50^ Finally, although the majority of opioid overdose fatalities can be attributed to the use of synthetic opioids such as fentanyl, high-risk opioid prescribing, including extended duration or high-dosage opioid prescription, is a factor that can contribute to increased risk of opioid misuse and overdose.

Much of the disparity in access to health insurance and MOUD can be attributed to differences in rural and urban environments. The discrepancy between rural and urban socioeconomic stressors is well understood, and access to health care and opioid use disorder treatment programs are scarcer in rural areas.^25^ Furthermore, when compared to rural communities, suburban communities have experienced higher opioid-related mortality rates since 2016.^51^ In addition, rural communities may be more affected by social and economic stressors caused by COVID-19. Understanding the dynamics of opioid misuse between communities with different levels of urbanicity during COVID-19 is key to constructing appropriate targeted interventions in Kentucky. Highlighting Appalachian counties in the context of opioid overdose in Kentucky is crucial—opioid overdose rates in Appalachia are significantly higher due to various factors specific to the region. Specifically, lack of transportation and health services, as well as economic deprivation, make the opioid epidemic in Appalachia uniquely severe compared to the rest of Kentucky.^52^,^53^

For all these reasons, it is crucial to understand the pandemic’s effect on opioid overdose in Kentucky. Within this state, large-scale societal changes following COVID-19 onset coupled with existing demographic and geographic disparities, and worsened opioid overdose outcomes. The present study aims to characterize the changes in opioid overdose fatality rates in Kentucky during the pandemic period (from 2019 to 2021), with a specific focus on changes among Appalachian v. non-Appalachian Kentucky residents. It also explores which factors were associated with opioid overdose fatalities in 2019 and 2021 and how these associations may have changed over time. It is hoped that findings will inform targeted interventions to improve both opioid overdose prevention and OUD treatment.

## METHODS

### Measures and Data Sources

The main outcome of interest was county-level yearly opioid overdose fatalities in Kentucky per 100,000 residents. Opioid overdose fatality counts were calculated using death certificates of Kentucky residents, extracted from the Kentucky Office of Vital Statistics. Opioid-involved overdose deaths for Kentucky residents over the age of 18 were identified by an underlying cause-of-death ICD-10 code in the range X40–X44, X60–X64, X85, Y10–Y14 and a supplementary ICD-10 cause-of-death code in the range T40.0–T40.4 or T40.6. To calculate fatality rates, estimates for Kentucky population over the age of 18 were obtained from the U.S. Census Bureau American Communities Survey (ACS) in 2019.^54^

Based on proposed links to opioid overdose presented in the literature, a variety of potential factors related to opioid overdose in Kentucky—including COVID-19-related factors, demographics, county metropolitan and Appalachian status, and factors related to MOUD—were analyzed.

For the COVID-19-related factors of interest, county-level variables related to unemployment and mental health were investigated. Monthly unemployment rates from both 2019 and 2021 were obtained from the National Bureau of Labor Statistics (BLS).^24^ The number of mental health providers per 100,000 residents, which was obtained from National Provider Information (NPI) data from 2019 provided by the Centers for Medicare & Medicaid Services (CMS), were used to account for the availability of mental health care in each county.^55^

A county was defined as Appalachian following the designation described by the Appalachian Regional Commission.^56^ To account for disparities between rural and urban communities, county metropolitan status was defined using Rural–Urban Continuum Codes from the U.S. Department of Agriculture’s Economic Research Service.^57^ County metropolitan status was divided into metropolitan (continuum codes 1–3), adjacent-to-metropolitan (continuum codes 4–6), and non-metropolitan groups (continuum codes 7–9).

Data on percentage non-white, percentage residents over the age of 65, and percentage residents in poverty were obtained from the U.S. Census Bureau American Communities Study in 2019.^54^ The percentage of residents in each county who were uninsured was obtained from the U.S. Census Bureau’s Small Area Health Insurance Estimates (SAHIE) program.^58^

Prevalence of OUD estimates in 2019 were calculated for each Kentucky county by Thompson et al.^59^ using Multiple Systems Estimation and were shared for this study. This method linked data from multiple Kentucky healthcare data sources, and the number of individuals with OUD on each combination of lists were used to estimate the number of individuals with OUD that were unobserved.^60^

Number of naloxone units distributed in Kentucky communities for both 2019 and 2021 was obtained from the Kentucky Pharmacists Association (KPhA).^61^ The Kentucky All Schedule Prescription Electronic Reporting (KASPER) program monitors all controlled substance prescriptions dispensed in Kentucky. Using KASPER data, the number of individuals aged 18 years or older receiving buprenorphine treatment for OUD was used to calculate the monthly rate of buprenorphine receipt in 2019.^62^ Methadone for treatment of OUD is dispensed at OTPs and not reported to the KASPER program. Thus, availability of methadone MOUD could not be accounted for. Finally, KASPER data were used to calculate measures for high-risk opioid prescribing in 2019.^62^

### Statistical Analysis

**Figure 1:**
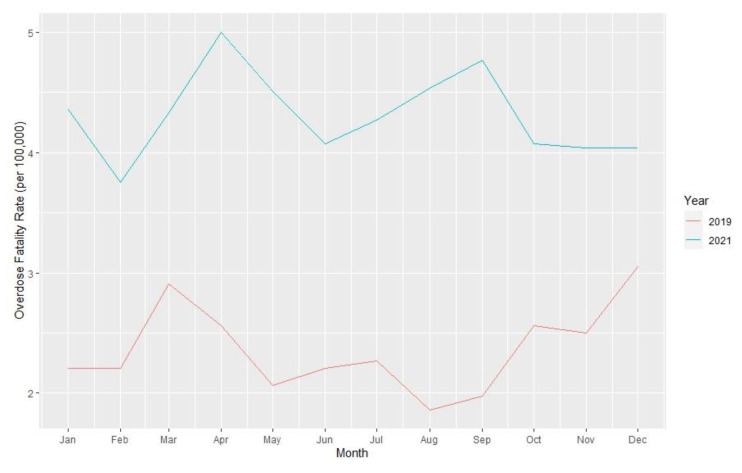
Opioid Overdose Fatality Rates per 100,000 Kentucky Residents in 2019 and 2021

**Figure 2:**
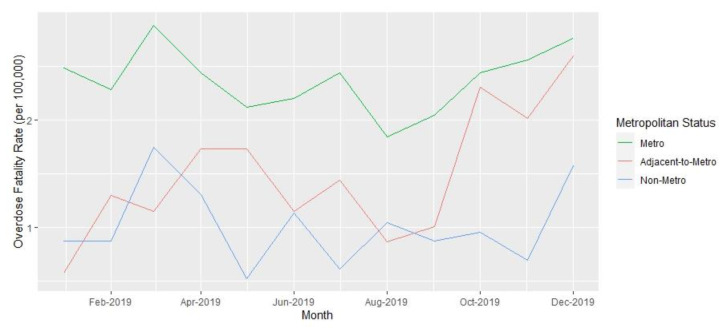
Opioid Overdose Fatality Rates in Kentucky by Metropolitan County Status, 2019

**Figure 3:**
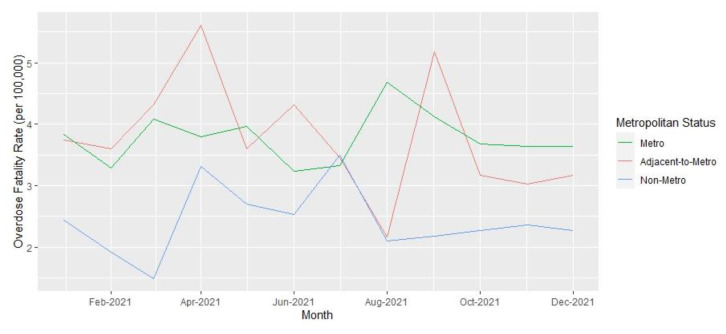
Opioid Overdose Fatality Rates in Kentucky by Metropolitan County Status, 2021 NOTE: Note for figure

**Figure 4:**
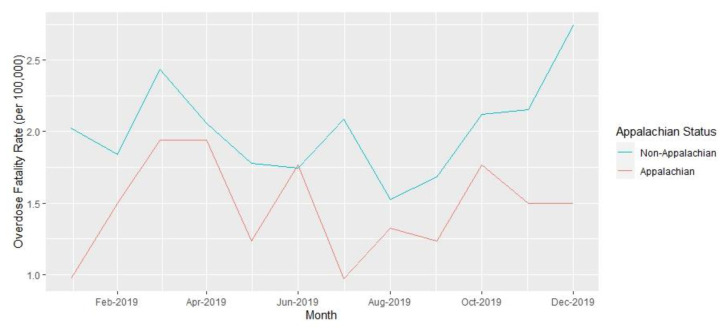
Opioid Overdose Fatality Rates in Kentucky by Appalachian Status, 2019

**Figure 5:**
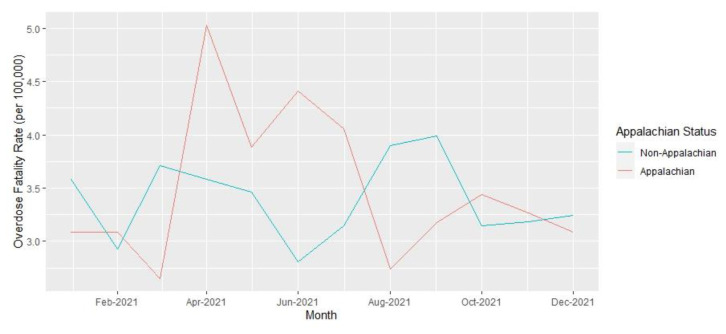
Opioid Overdose Fatality Rates in Kentucky by Appalachian Status, 2021

Analyses were performed at the county level. Yearly opioid overdose fatality rates and 95% confidence intervals per 100,000 residents over age 18 years for 2019 and 2021, as well as the rate ratios comparing rates between the two years, were calculated. Opioid fatality counts and rates for 2019 and 2021, with 95% confidence intervals, are presented in Table 1 and are stratified by Appalachian county status and metropolitan classification. County-level summary statistics for 2019 variables are presented in Table 2. Unemployment rates, naloxone distribution rates, and rate ratios for these variables over the two years are presented in Table 3. Monthly opioid overdose rates for 2019 and 2021 are presented in Figure 1 and are stratified by metropolitan and Appalachian county status for each year in Figures 2–5.

To determine which county-level factors were associated with opioid overdose fatality rates in 2019 and 2021, as well as if these associations changed, an adjusted marginal generalized estimating equation (GEE)-type negative binomial model was fit.^63^,^64^ The outcome for each county in a given year was defined as the number of opioid overdose fatalities, and the statistical correlation among count outcomes from the same county was modeled using working unstructured covariance matrices. Due to variation in county population, the model’s offset was the natural log of the number of residents in a given county. Yearly rates were directly modeled, and rate ratios were used as the basis for comparisons between years. Results for the associations for each year as well as whether the changes in association between 2019 and 2021 are statistically significant are presented Table 3. Estimates for the changes in associations are presented in Table 4 and can be interpreted as the ratio of the rate ratios for associations in 2021 vs. 2019. Analyses were conducted using SAS version 9.4 (SAS Institute, Cary, NC, USA) and R version 3.6.1 (R Foundation for Statistical Computing, Vienna, Austria). Statistical significance was defined as *p* < .05.

## RESULTS

### Descriptive Results

As presented in Table 1, opioid overdose fatalities increased from 976 in 2019 to 1780 in 2021 (RR: 1.82). Appalachian counties had a lower fatality rate than non-Appalachian counties in 2019 (22.01 v. 33.06 per 100,000 residents) but experienced a larger increase (RR: 2.38 v. 1.68) in fatality rate from 2019 to 2021. The fatality rate in Appalachian counties was higher than that of non-Appalachian counties in 2021 (52.26 v. 51.56 per 100,000 residents).

Among the three metropolitan status categories, adjacent-to-metropolitan counties experienced the largest increase in opioid overdose fatality rates from 2019 to 2021, with a rate ratio of 2.54. In contrast, metropolitan had the highest fatality rate in 2019, with 35.19 fatalities per 100,000 residents but experienced the lowest increase in fatalities from 2019 to 2021, with a rate ratio of 1.59.

The county-level average for unemployment rate increased from 4.82% to 5.16% (RR: 1.07) from 2019 to 2021 (Table 2). The naloxone distribution rate per 1,000 residents increased drastically from 2019 to 2021 (RR: 3.01).

In 2019, the average county percentage of residents older than 65 years was 17.44%; the average county percentage of non-white residents was 6.93%, and the percentage living in poverty was 19.73% (Table 2). The baseline assessment of opioid use disorder prevalence was 53.53 per 1,000 residents. In 2019, on average, every month 1.72 per 1,000 residents had dispensed buprenorphine prescription(s) for treatment of opioid use disorder; 1.86 per 1,000 residents met the criteria for high-risk opioid prescribing.

### Modeling Results

Regression results for 2019 and 2021 are displayed in Table 3, and results for the change in rates are displayed in Table 4. After accounting for other variables in the model, we found no significant difference in the opioid overdose fatality rate between Appalachian and non-Appalachian counties in 2019 (RR: 1.12, 95% CI: (0.72, 1.77)) and in 2021 (RR: 1.55, 95% CI: (1.04, 2.32)) (Table 3). The change in association between Appalachian status and opioid overdose mortality from 2019 to 2021 was not statistically significant (p-value: 0.136).

In 2019, metropolitan county status, compared to non-metropolitan county status, was associated with an increase in opioid overdose fatalities (RR: 1.96, 95% CI: (1.25, 3.07), *p* = .003).This association had a statistically significant decrease from 2019 to 2021 (RR: 0.61, 95% CI: (0.40, 0.94), *p* = .023), showing shrinking differences in mortality between metropolitan and non-metropolitan counties. Metropolitan county status did not have a statistically significant association with opioid overdose fatalities in 2021. On the other hand, in 2021, adjacent-to-metropolitan counties had an opioid overdose mortality rate almost two times that of non-metropolitan counties (RR: 1.94, 95% CI: (1.30, 2.89), *p* = .001), while in 2019 the difference in their rates was not significant (RR 1.48, 95% CI: (0.85, 2.59)).

Neither of the COVID-19-related variables—unemployment or mental health providers—had clinically or statistically significant associations in either year, nor were the changes in associations from 2019 to 2021 statistically significant. As for the medication-related variables, the associations between estimated opioid use disorder prevalence in 2019 and overdose fatalities in both years were statistically significant (2019 – RR: 1.01, 95% CI: (1.00, 1.02), *p* = .002 / 2021 – RR: 1.01, 95% CI: (1.00, 1.02), *p* = .008), but the change in associations between the years was not statistically significant (*p* = .626). None of the other medication-related variables were associated with opioid overdose fatalities in either year.

Of the demographic variables, the 2019 percentage residents over age 65 years was statistically significantly associated with opioid overdose fatalities in both 2019 (RR: 0.94, 95% CI: (0.91, 0.98), *p* = .002) and 2021 (RR: 0.88, 95% CI: (0.85, 0.92), *p* < .001). In addition, the change in association from 2019 to 2021 was statistically significant (RR: 0.94, 95% CI: (0.91, 0.97), *p* < .001). None of the other demographic variables were statistically significantly associated with opioid overdose in either year.

## DISCUSSION

The analysis shows an increased gap in opioid overdose mortality between Appalachian and non-Appalachian counties from 2019 to 2021 (adjusted rate ratios 1.12 v. 1.55). This may indicate that the pressures brought on by the COVID-19 pandemic affected disparately Appalachian counties. Appalachian counties are vulnerable to opioid overdose due to issues such as economic deprivation and lack of access to health care, which became more prevalent during the pandemic.^20^,^23^ Proliferation of alternative solutions to tackling opioid overdose treatment in Appalachia, such as telehealth, is crucial even beyond COVID-19 due to persistent barriers such as spatial access to health care.^65^ The increase in naloxone distribution rate from 2019 to 2021 (RR: 3.01) may be attributed to recent efforts to expand naloxone distribution in Kentucky, such as a Kentucky General Assembly Amendment in June 2019, which expanded the ability of pharmacies to distribute naloxone.^66^

When comparing metropolitan county status with non-metropolitan status, the adjusted rate ratio decreased from 2019 to 2021 (1.96 v. 1.21). This may be due to the societal changes brought about by COVID-19 disproportionately affecting non-metropolitan counties. Increased psychological distress combined with the lack of access to appropriate health care could have been a contributor to an increased rate of opioid overdose in rural communities.^20^,^25^ However, when comparing adjacent-to-metropolitan county status with non-metropolitan status, the adjusted rate ratio increased from 2019 to 2021 (1.48 v. 1.94). One possible explanation for this result is that due to occupations commonly found among suburban residents transitioning to telework during the pandemic, time spent in isolation may have increased, resulting in poorer mental health over the course of COVID-19. Further investigation into the environment of suburban Kentucky during the COVID-19 pandemic is necessary for clarification on this result.

Percentage residents over aged 65 years was associated with a decrease in opioid overdose fatality rates for both 2019 and 2021. Notably, this association was stronger in 2021 than in 2019. Since opioid overdose is generally more common among younger populations, it is possible that younger communities’ vulnerability to the societal pressures caused by COVID-19 increased their susceptibility to opioid overdose. Kentucky residents aged 15–24 years experienced the highest increase (81%) in drug overdose fatality rates from 2019 to 2021.^15^

There are several strengths of this study. Previous studies have examined the association between demographic and socioeconomic factors and opioid overdose, as well as opioid overdose during COVID-19; however, this study is unique in its simultaneous examination of both factors involved in societal changes during the COVID-19 pandemic and how these associations changed over time. In addition, there is a lack of research regarding long-term opioid overdose trends after the COVID-19 pandemic. While there exists some literature examining the short-term implications of COVID-19 on opioid overdose, 2021 data have not been extensively studied despite the fact that they can provide insights on the lasting effects of the pandemic. Being able to construct interventions and programs in a post-COVID-19 world is crucial to improving treatment and preventive measures in the current landscape.

Despite the strengths of this study, there are some limitations. Social isolation became more prevalent during the onset of the COVID-19 pandemic due to government-imposed measures to enforce social distancing. Although social isolation has been linked to opioid overdose, it was not accounted for in this analysis. Social isolation also presents a unique roadblock in traditional forms of opioid overdose reversal, as unconscious individuals experiencing an overdose event will not be able to administer naloxone. Social isolation has been linked to poor mental health and psychological stress, which are both factors associated with increased risk of opioid overdose.^67^,^68^ Isolation may also increase sensitivity to chronic pain, which may lead individuals to seek opioids as pain treatment.^69^ It would be fruitful for future studies to analyze social isolation as a factor associated with opioid overdose fatalities. Social isolation caused by unemployment could have also contributed to unemployment’s lack of statistically significant associations with overdose fatalities in either year.

In addition, it was not possible to account for changes in several of the factors during the pandemic, as 2021 data were not available for all examined factors. For example, county-level data describing the proliferation of telehealth in Kentucky and loosening of buprenorphine prescription restrictions were not included in the models.^70^ This may have contributed to the lack of detected change in associations from 2019 to 2021. Incorporating 2021 data into the analysis would allow one to investigate associations in 2021, as well as the changes in associations between the two years, more precisely. Furthermore, factors that contribute to a county’s vulnerability to COVID-19, such as population density and social distancing measures, were unable to be accounted for. These factors may have been associated with opioid overdose, as vulnerability to COVID-19 may be representative of vulnerability to other adverse health events, such as opioid overdose. The COVID-19 Pandemic Vulnerability Index (PVI) is a measure developed by the National Institute of Environmental Health Sciences (NIEHS) to represent each county’s vulnerability to COVID-19.^71^ However, since this score is composite and contains factors already accounted for in our model, such as demographic information, it was not included. Future studies could investigate variables like those that compose the PVI to understand how factors that contribute to a county’s vulnerability to COVID-19 affected opioid overdose rates. Finally, the opioid use disorder prevalence variable used in this analysis was an estimate as opposed to the true, unknown value, and hence estimated associations may be biased.

A lack of variability in community-level covariates can imply a lack of power. This lack of sufficient variability manifests itself as two major limitations to the analysis: First, because certain demographic variables that could be confounders, such as gender, do not vary significantly between counties, they were not included. Second, several of the factors which have been previously linked to opioid overdose were not found to be associated with overdose fatalities in the analysis. For example, unemployment rate has been found to be associated with opioid overdose and substance misuse in general.^25^,^26^ However, here unemployment rate was not found to be associated with opioid overdose fatalities in either 2019 or 2021. Unemployment rate’s relatively small range among counties in both years (7.4% for 2019 and 9.1% in 2021) is indicative of low variability. Thus, the ability to detect a statistically significant association between unemployment rate and opioid overdose fatalities was not as high as if individual-level data had been available and used.

Finally, there are some limitations to the measures chosen to be included in the model. Many of the measures may not be accurate representations of the factor in question. For example, while mental health providers per 1,000 residents provides a picture of the available resources to treat mental health, it does not elucidate the entirety of the mental health situation for each county.

## IMPLICATIONS

Despite the aforementioned limitations, this study provides salient information on how various factors could be associated with opioid overdose before and during the COVID-19 pandemic. Opioid overdose fatality rates increased in Kentucky from 2019 to 2021, with the largest increases occurring in adjacent-to-metropolitan and Appalachian regions. Several variables, such as metropolitan status and Appalachian status, as well as age, were found to be associated with opioid overdose fatality rates in one or both of the years. In addition, metropolitan status and age were found to have observed associations that changed from 2019 to 2021. These findings can be used to construct targeted interventions that improve treatment and prevention in adjacent-to-metropolitan areas, as well as amongst younger populations. Future studies should aim to investigate other COVID-19-related variables as well as which factors are associated with overdose fatality rates in other U.S. regions.

**SUMMARY BOX**

**What is already known about this topic?**

Kentucky has one of the highest rates of opioid overdose fatalities in the U.S., and these rates have further increased during the COVID-19 pandemic. Several potential factors associated with this increase that have been exacerbated by the pandemic, such as poor mental health and access to health care.

**What is added by this report?**

Opioid overdose fatality rates drastically rose in Kentucky from 2019 to 2021, with adjacent-to-metropolitan and Appalachian counties experiencing the largest increases. Several county-level factors, such as age and metropolitan county status, have been observed to be associated with opioid overdose fatalities in Kentucky in 2019 and 2021.

**What are the implications for future research?**

Metropolitan status’s observed association with opioid overdose fatalities in both years can be indicative of a discrepancy of the impact of the COVID-19 pandemic on access to opioid overdose treatment between metropolitan and non-metropolitan communities. These findings can be used to drive targeted interventions to improve treatment of opioid overdose in Kentucky, specifically in non-metropolitan areas. Future research can investigate other factors that may be associated with overdose fatalities as well as investigate other U.S. regions.

## Figures and Tables

**Table 1:** Opioid Overdose Fatality Counts and Rates per 100,000 Residents in Kentucky in 2019 and 2021

Fatality Counts (Rates per 100,000 Residents)			
	2019	2021	RR* (95% CI†)
**Overall**	976 (28.37)	1780 (51.75)	1.82 (1.68, 1.97)
**Metropolitan Status**			
Metropolitan	712 (35.19)	1132 (55.96)	1.59 (1.45, 1.75)
Adjacent-to-Metropolitan	124 (23.51)	315 (59.73)	2.54 (2.06, 3.13)
Non-Metropolitan	164 (18.78)	348 (45.88)	2.44 (2.12, 2.81)
**Appalachian Status**			
Appalachian	200 (22.01)	475 (52.26)	2.38 (2.02, 2.81)
Non-Appalachian	776 (30.66)	1305 (51.56)	1.68 (1.54, 1.84)

NOTES:

* RR = Rate Ratio

† CI = Confidence Interval

**Table 2:** County-level Means of Variables from 2019 and 2021

	2019		2021		RR
	Mean (SD*)	95% CI	Mean (SD)	95% CI	2021 v. 2019
**Mental Health Providers (per 1,000 Residents)**	1.67 (2.70)	(1.19, 2.16)	-	-	-

**Demographic Variables**					

% Age > 65	17.44 (3.36)	(16.84, 18.05)	-	-	-

% Non-White	6.93 (5.49)	(5.94, 7.91)	-	-	-

% Poverty	19.73 (6.63)	(18.55, 20.92)	-	-	-

Uninsured % (% of population under 65 without health insurance)	6.01 (0.91)	(5.89, 6.13)	-	-	-

**Medication-Related Variables**					

Monthly Buprenorphine Reception Rate (per 1,000 Residents)	1.72 (1.35)	(1.55, 1.89)	-	-	-

Monthly High-Risk Opioid Prescribing Rate (per 1,000 Residents)	1.86 (0.53)	(1.80, 1.93)	-	-	-

Opioid Use Disorder Prevalence (per 1,000 Residents)	53.53 (32.52)	(49.40, 59.35)	-	-	-

Unemployment Rate (%)	4.82 (1.31)	(4.59, 5.05)	5.16 (1.41)	(4.91, 5.41)	1.07 (1.06, 1.08)

Naloxone Distribution Rate (per 1,000 Residents)	4.98 (9.49)	(4.82, 5.13)	14.99 (22.19)	(14.63, 15.36)	3.01 (2.96, 3.06)

NOTES:

* SD = Standard Deviation

**Table 3:** Model Estimates (Adjusted Rate Ratios) for Yearly Opioid Overdose Fatalities per 100,000 Residents

Variable	2019 RR (95% CI)	2021 RR (95% CI)
**COVID-19-Related Variables**		
Unemployment Rate	0.99 (0.87, 1.14)	0.97 (0.89, 1.06)
Mental Health Providers per 1,000 Residents	0.99 (0.96, 1.03)	1.02 (0.99, 1.05)
**Geographical Variables**		
***Metropolitan Status***		
Metropolitan v. Non-Metropolitan†	1.96 (1.25, 3.07) *	1.21 (0.78, 1.86)
Adjacent-to-Metropolitan v. Non-Metropolitan	1.48 (0.85, 2.59)	1.94 (1.30, 2.89) *
Metropolitan v. Adjacent-to-Metropolitan†	1.33 (0.75, 2.35)	0.62 (0.42, 0.92) *
***Appalachian Status***		
Appalachian v. Non-Appalachian	1.12 (0.72, 1.77)	1.55 (1.04, 2.32) *
**Demographic Variables**		
Non-white %	0.99 (0.96, 1.03)	1.00 (0.98, 1.03)
Age > 65 %†	0.94 (0.91, 0.98) *	0.88 (0.85, 0.92) *
Poverty %	0.98 (0.95, 1.02)	0.99 (0.96, 1.01)
Uninsured % (% of population under 65 without health insurance)	0.98 (0.83, 1.16)	0.96 (0.85, 1.09)
**Medication-Related Variables**		
Monthly Naloxone Distribution per 1,000 Residents	1.01 (0.99, 1.02)	1.00 (1.00, 1.01)
Monthly Buprenorphine Reception Rate per 1,000 Residents	1.05 (0.83, 1.33)	1.04 (0.86, 1.27)
Monthly High-Risk Opioid Prescribing Rate per 1,000 Residents	0.88 (0.54, 1.45)	0.72 (0.49, 1.06)
Opioid Use Disorder Prevalence per 1,000 Residents	1.01 (1.00, 1.02) *	1.01 (1.00, 1.02) *

NOTES:

* *p* < .05

† Change in association between 2019 and 2021 is statistically significant. Results are presented in Table 4.

**Table 4:** Model Estimates (Adjusted Rate Ratios) for the Interactions Between Factors of Interest and Year for Yearly Opioid Overdose Fatalities per 100,000 Residents

Variable	Change, 2019 to 2021 RR* (95% CI^†^)	*p*-value
**COVID-19-Related Variables**		
Unemployment Rate	0.98 (0.87, 1.10)	0.675
Mental Health Providers per 1,000 Residents	1.03 (0.99, 1.07)	0.211
**Geographical Variables**		
*** Metropolitan Status***		
Metropolitan v. Non-Metropolitan	0.61 (0.40, 0.94)	0.023 *
Adjacent-to-Metropolitan v. Non-Metropolitan	1.31 (0.85, 2.02)	0.218
Metropolitan vs. Adjacent-to-Metropolitan	0.47 (0.31, 0.71)	<0.001 *
*** Appalachian Status***		
Appalachian v. Non-Appalachian	1.38 (0.90, 2.11)	0.136
**Demographic Variables**		
Non-white %	1.01 (0.99, 1.03)	0.291
Age > 65 %	0.94 (0.91, 0.97)	< 0.001 *
Poverty %	1.00 (0.97, 1.04)	0.814
Uninsured % (% of population under 65 without health insurance)	0.98 (0.85, 1.13)	0.751
**Medication-Related Variables**		
Monthly Naloxone Distribution per 1,000 Residents	1.00 (0.99, 1.01)	0.501
Monthly Buprenorphine Reception Rate per 1,000 Residents	0.99 (0.83, 1.18)	0.900
Monthly High-Risk Opioid Prescribing Rate per 1,000 Residents	0.82 (0.55, 1.21)	0.311
Opioid Use Prevalence per 1,000 Residents	1.00 (1.00, 1.01)	0.626

NOTES:

* *p* < .05
